# Reply to Cheng et al.: COVID-19 induces lower extent of cytokines, but damages vascular endothelium by IL-6 signaling

**DOI:** 10.1073/pnas.2105040118

**Published:** 2021-05-10

**Authors:** Sujin Kang, Toshio Tanaka, Hitomi Inoue, Chikako Ono, Shoji Hashimoto, Yoshiyuki Kioi, Hisatake Matsumoto, Hiroshi Matsuura, Tsunehiro Matsubara, Kentaro Shimizu, Hiroshi Ogura, Yoshiharu Matsuura, Tadamitsu Kishimoto

**Affiliations:** ^a^Department of Immune Regulation, Immunology Frontier Research Center, Osaka University, Osaka 565-0871, Japan;; ^b^Medical Clinic, Kinki Central Hospital, Hyogo 664-8533, Japan;; ^c^Department of Molecular Virology, Research Institute for Microbial Diseases, Osaka University, Osaka 565-0871, Japan;; ^d^Department of Clinical Laboratory, Osaka Habikino Medical Center, Osaka 583-8588, Japan;; ^e^Department of Traumatology and Acute Critical Medicine, Osaka University Graduate School of Medicine, Osaka University, Osaka 565-0871, Japan

We appreciate the constructive comments by Cheng et al. (1), who performed an intensive statistical analysis of cytokine levels in patients with COVID-19. Their work applies the inverse-variance weighted (IVW) method and an MR-Egger regression on a large number of patients for a statistical analysis to identify COVID-19 risk factors. Consistent with our previous work (2), the results of these analyses suggest that the plasma from patients with COVID-19 has significantly lower levels of interleukin (IL)-8, IL-10, and monocyte chemoattractant protein (MCP)-1, compared with that from patients with other types of cytokine release syndrome (CRS).

Previously, we proposed that the elevation of four proinflammatory cytokines, that is, IL-6, IL-8, IL-10, and MCP-1, in combination with the elevation of coagulation cascade activator plasminogen activator inhibitor-1 (PAI-1) is a common feature of different types of CRS including bacterial sepsis and acute respiratory distress syndrome (ARDS) (2). To understand the pathogenesis of COVID-19−induced CRS, we analyzed the spectrum of elevated cytokines in seven patients who were critically ill with COVID-19 and in healthy controls. We identified that levels of PAI-1 were comparable with those of other CRS types, and are highly correlated with severity of COVID-19. In contrast, the levels of the proinflammatory cytokines including IL-6, IL-8, IL-10, and MCP-1 in COVID-19 cases were not high in comparison with those observed in bacterial CRS cases. Additionally, we identified that IL-6 trans-signaling−induced PAI-1 signaling plays a critical role in the pathogenesis of critically ill COVID-19 patients. This mechanism can explain the death of COVID-19 patients who presented with endotheliitis and thrombosis, and it supports observations that treatment with anti−IL-6 receptor monoclonal antibody, tocilizumab, can be effective for such patients.

The IL-6 concentrations in patients with COVID-19 are significantly lower than those in patients with sepsis or ARDS patients (2, 3). COVID-19 patients were heterogeneous, from critical to severe cases, in the primary analysis, with a broad range of IL-6 concentrations, from 6.5 pg/mL to 357.2 pg/mL (3). Notably, severe acute respiratory syndrome coronavirus 2 infection causes pulmonary endothelial injury and increases IL-6 production by endothelial cells (4). Moreover, several in vitro data from multiple studies indicate that IL-6 trans-signaling in vascular endothelial cells can induce the production of IL-6, IL-8, and MCP-1. The extent to which severe COVID-19 causes endothelial injury can depend on the site of insult and the basis of systemic inflammatory profiles (5, 6). Importantly, the levels of acute-phase proteins such as C-reactive protein, which is induced by IL-6, and D-dimer were elevated in patients with COVID-19, and the concentrations of these proteins were comparable between patients with COVID-19 and those with sepsis. Increasing IL-6 levels might function in endothelial activation and the coagulation cascade in pulmonary tissue; this possibility supports the findings of recent clinical trials indicating that IL-6 signaling-inhibiting therapeutics are beneficial for treating severe cases of COVID-19 (7, 8).
